# Work rate adjustments needed to maintain heart rate and RPE during high-intensity interval training in the heat

**DOI:** 10.3389/fphys.2025.1506325

**Published:** 2025-02-06

**Authors:** Hillary A. Yoder, Anne M. Mulholland, Hayley V. MacDonald, Jonathan E. Wingo

**Affiliations:** ^1^ Department of Kinesiology, The University of Alabama, Tuscaloosa, AL, United States; ^2^ Department of Kinesiology, New Mexico State University, Las Cruces, NM, United States; ^3^ Department of Exercise Science, Mercer University, Macon, GA, United States

**Keywords:** exercise prescription, HIIT, cardiovascular drift, power output, heat stress, target heart rate, rating of perceived exertion

## Abstract

**Introduction:**

Higher work rates may be sustainable when maintaining target rating of perceived exertion (RPE) compared to maintaining target heart rate (THR) during high-intensity interval training (HIIT) exercise in hot conditions, but may also result in greater thermal strain and cardiovascular drift, as well as greater decrements in maximal oxygen uptake ( $\dot{V}$ O_2max_).

**Purpose:**

To test the hypotheses that maintaining target RPE compared to THR during HIIT in the heat results in 1) smaller work rate adjustments, 2) greater thermal and cardiovascular strain, and 3) larger decreases in $\dot{V}$ O_2max_.

**Methods:**

Eight adults (4 women) completed a graded exercise test on a cycle ergometer in 22°C and then 4 cycling trials in 35°C, consisting of an 8-min warm-up at 70% maximal heart rate (HR_max_) or 12 RPE followed by 1 (15_HR_ and 15_RPE_) or 5 (43_HR_ and 43_RPE_) rounds of HIIT (1 round = 4 min work at 90% HR_max_ or 17 RPE and 3 min recovery at 70% HR_max_ or 12 RPE) totaling 15 min or 43 min of exercise, respectively. Each trial ended with a GXT to measure $\dot{V}$ O_2max_.

**Results:**

In the 43-min trials work rate decreased from the first to the fifth work interval in both conditions, but by a non-significant, yet moderately larger (ES = 0.53) amount during 43_HR_ (46 ± 29 W) compared to 43_RPE_ (30 ± 28 W). From the first to fifth work interval HR increased over time by 12 b⋅min^–1^ in 43_RPE_ (*p* < 0.001), but did not increase during 43_HR_ (*p* = 0.36). Rectal temperature increases were not different between conditions (43_HR_ = 0.7°C, *p* < 0.001; 43_RPE_ = 0.8°C, *p* < 0.001). $\dot{V}$ O_2max_ decreased 15.6% (ES = 0.41) between 15_RPE_ and 43_RPE_ (*p* = 0.005), but it was not different over time during the HR-based trials [6.5%, ES = 0.16 (α adjusted for multiple comparisons = 0.0125) *p* = 0.03].

**Conclusion:**

Maintaining target RPE and THR require considerable declines in work rate during HIIT in the heat, with ∼53% larger declines needed to maintain THR. The mitigation of cardiovascular drift in the THR trial may have contributed to the preservation of $\dot{V}$ O_2max_.

## 1 Introduction

The intensity of work and rest intervals during high-intensity interval training (HIIT) can be prescribed using work rate (speed or power output), oxygen uptake ( $\dot{V}$ O_2_), metabolic equivalents, heart rate (HR), or rating of perceived exertion (RPE). Each method has different advantages and disadvantages. For instance, using work rate can be problematic because a single velocity can represent varying metabolic demands depending on the terrain and environment, and some speeds may not be attainable in certain conditions such as high winds, steep hills, or oppressive heat. Furthermore, outside of a laboratory, prescribing intensity using $\dot{V}$ O_2_ is impractical because of expensive and cumbersome equipment needed to measure $\dot{V}$ O_2_ directly and general unfamiliarity with using metabolic equations if $\dot{V}$ O_2_ is to be estimated.

Because of these limitations, and as a result of its ease of use and linear relationship with $\dot{V}$ O_2_ (Swain et al., 1994), target HR (THR) is often used for prescribing intensity of HIIT (Morales-Palomo et al., 2017; Arazi et al., 2017; Helgerud et al., 2007). However, using THR to gauge exercise intensity is complicated by a phenomenon known as cardiovascular drift, whereby a progressive increase in HR occurs over time despite no change in work rate. Under conditions in which cardiovascular drift occurs, work rate must be lowered to maintain THR, which can compromise the training stimulus and, subsequent adaptations (Wingo, 2015; Morales-Palomo et al., 2017; Wingo and Cureton, 2006b; Yoder et al., 2023). Historically cardiovascular drift has been applied to conditions of prolonged, continuous, moderate-intensity exercise but more recently it has been observed during HIIT in temperate (24°C) and hot (35°C) environments (Morales-Palomo et al., 2017). Using THR to prescribe exercise intensity during HIIT in hot conditions was shown to be especially problematic, necessitating 33% work rate decrements over 43 min of exercise (Yoder et al., 2023).

A simple alternative to using THR when prescribing intensity of a HIIT session is to use rating of perceived exertion (RPE), a subjective measure of intensity (Borg and Noble, 1974). RPE is an appealing method of prescribing exercise intensity because it requires no equipment, allows the individual to adjust the intensity based on how the intensity of exercise is perceived, and in young healthy individuals, is directly related to HR (Borg, 1982). RPE has been repeatedly shown to be a valid method to gauge exercise intensity in temperate conditions (Eston and Williams, 1988; Edwards et al., 1972; Dunbar et al., 1992). During constant-intensity exercise in the heat, however, RPE is elevated compared to cooler environments (Maw et al., 1993), and, like HR, it may progressively increase over time despite no change in work rate (Pandolf, 1998; Wingo and Cureton, 2006a; Wingo et al., 2005; Wingo et al., 2020). Therefore, like THR, to maintain target RPE in hot environments, work rate must be lowered to a larger extent compared to that in cooler environments (Tucker et al., 2006; Roussey et al., 2018). Even so, the magnitude by which work rate needs to be lowered to maintain target RPE in the heat appears to be less than that to maintain THR (Tucker et al., 2006). Consequently, using target RPE to gauge exercise intensity during HIIT in the heat may be advantageous compared to using THR because a higher work rate can be maintained and thereby a greater training stimulus, but this has not been evaluated. An unintended, but important consequence of this, will likely be higher core body temperature and amplified cardiovascular strain (indexed as cardiovascular drift), but no study has addressed the extent to which this may occur.

In addition to the aforementioned considerations regarding cardiovascular drift, a consequence of cardiovascular drift is that it corresponds to reduced maximal oxygen uptake ( $\dot{V}$ O_2max_) during continuous exercise in hot conditions (Wingo et al., 2005; Lafrenz et al., 2008). This has implications for how exercise is perceived (e.g., if $\dot{V}$ O_2max_ declines during an exercise bout, a given work rate momentarily represents a greater proportion of $\dot{V}$ O_2max_, and therefore, will be perceived as more taxing). Since elevated core and skin temperatures and accompanying cardiovascular drift are associated with declines in $\dot{V}$ O_2max_ (Wingo et al., 2005; Nybo et al., 2001; Cheuvront et al., 2010), and since HIIT prescribed using RPE is expected to result in higher core and skin temperatures—and thereby greater cardiovascular drift—then HIIT based on RPE is expected to also result in larger declines in $\dot{V}$ O_2max_ compared to HIIT prescribed using HR, but this has not been tested.

Given the preceding notions, the purposes of this study were to test the hypotheses that 1) work rate would be lowered to a greater extent to maintain THR than to maintain target RPE during HIIT in a hot environment, 2) greater thermal and cardiovascular strain would result from maintaining target RPE compared to THR during a HIIT workout in the heat, and 3) $\dot{V}$ O_2max_ would decrease to a greater extent after HIIT in the heat when exercise intensity during HIIT is based on target RPE compared to THR.

## 2 Materials and methods

### 2.1 Experimental design

Participants visited the laboratory on 5 separate days (1 control trial, two 15-min experimental trials, and two 43-min experimental trials). At each visit, they completed an exercise bout on a cycle ergometer (LC6 Novo, Monark Exercise, Vansbro, Sweden).

**FIGURE 1:**
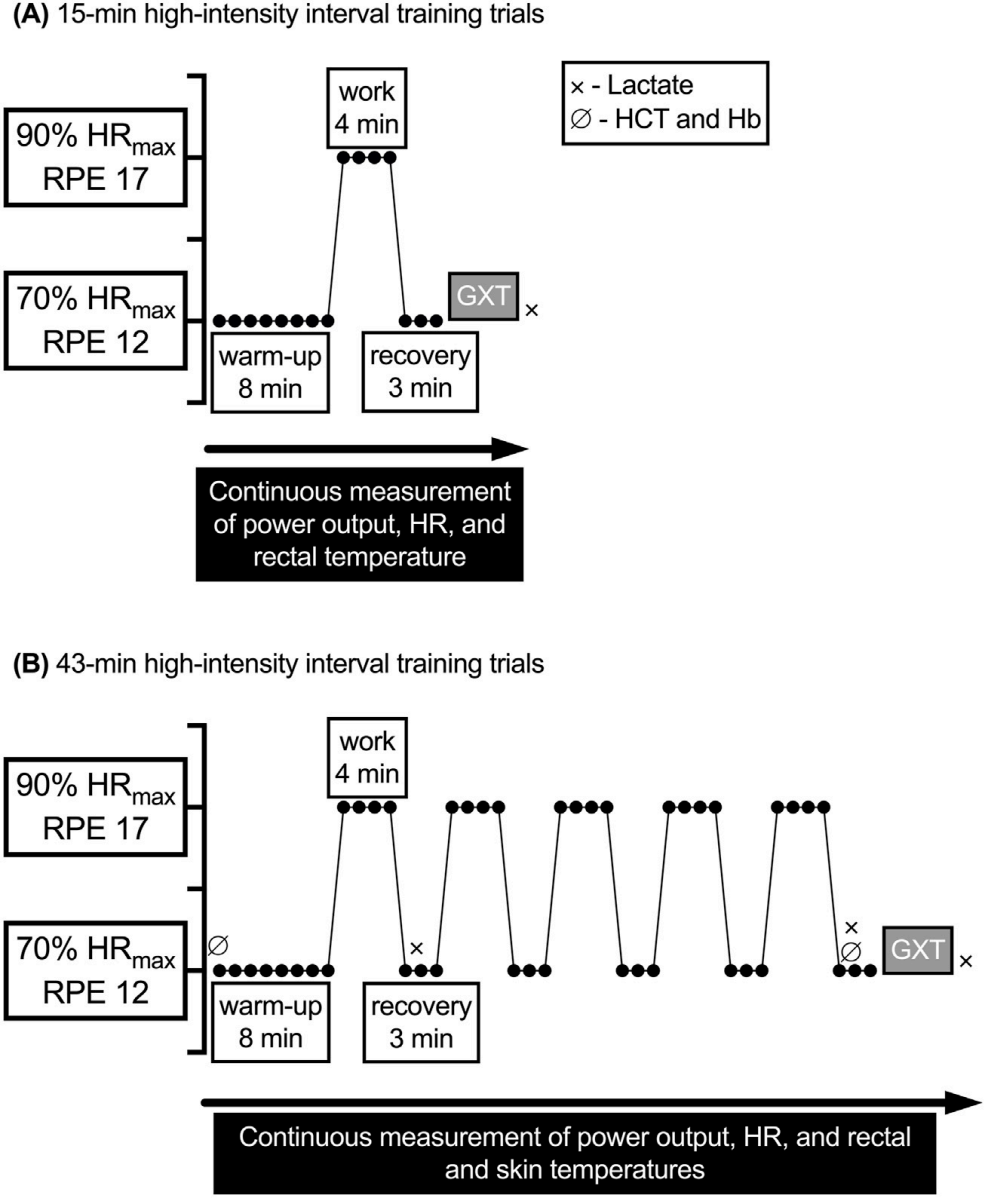
General exercise protocols for the 15- and 43-min experimental trials. GXT, graded exercise test; Hb, hemoglobin: HCT, hematocrit; HR, heart rate; HRmax, maximal heart rate; RPE, rating of perceived exertion; $\dot{V}$ O_2_, oxygen uptake. **(A)** 15-min high-intensity interval training trials. **(B)** 43-min high-intensity interval training trials.

The first visit was a control trial; participants completed a graded exercise test (GXT) to measure maximal HR (HR_max_) and $\dot{V}$ O_2max_ in a temperate environment [22.6°C ± 0.6°C, 36.6% ± 5.8% relative humidity (RH)]. The remaining 4 experimental trials were completed in a counterbalanced order and a hot environment (35.1°C ± 0.3°C, 40% ± 4% RH). Counterbalanced treatment orders were randomly assigned to participants. Each experimental trial consisted of an 8-min warm-up at 70% HR_max_ or an RPE of 12, followed by 1 (15_HR_ and 15_RPE_) or 5 (43_HR_ and 43_RPE_) rounds of HIIT using HR or RPE to prescribe the intensity. One round of HIIT consisted of 4 min at 90% HR_max_ or RPE of 17 and 3 min at 70% HR_max_ or RPE of 12 (Figure 1). If 70% HR_max_ or an RPE of 12 could not be achieved during the rest intervals (because of thermal and cardiovascular strain), participants cycled at 30 W with a cadence ≥30 rev⋅min^−1^; 30 W was selected to ensure participants were not recovering passively and were still cycling against resistance. Upon the completion of each experimental trial, without cessation of exercise, participants immediately began a GXT, performed at approximately half of the maximal power output observed during the control trial, to measure $\dot{V}$ O_2max_. Because cardiovascular drift typically occurs after 10–15 min of exercise, necessitating work rate adjustments to maintain THR or RPE, the purpose of the separate 15- and 43-min trials was to evaluate $\dot{V}$ O_2max_ before (15-min trials) and after (43-min trials) work rate adjustments had been made in order to maintain the prescribed intensities. Additionally, the 15-min trials were needed because it is not feasible to measure $\dot{V}$ O_2max_ at 15 min and 43 min within the same trial.

An *a priori* power analysis (G*power 3.1.9.6) revealed a sample size of 7 would be sufficient to detect a 25-W difference between the change score in power output from the first to the final work interval in 43_HR_ *versus* 43_RPE_, assuming α = 0.05 and power ≈0.80 (Faul et al., 2009; Faul et al., 2007). Eight healthy adults (4 men and 4 women; 18–38 y) free of disease participated. Seven were recreationally active as defined by the American College of Sports Medicine (2022) (i.e., exercising at a moderate intensity aerobically ≥30 min per day, ≥3 times per week, for the past ≥3 months) and 1 male was a competitive endurance athlete. Physical characteristics of participants were age (mean ± SD) = 25 ± 7 y, body mass = 74.1 ± 8.3 kg, height = 181 ± 10 cm, percent body fat = 21.4% ± 8.4%, $\dot{V}$ O_2max_ = 3.2 ± 1.2 L⋅min^–1^, HR_max_ = 185 ± 5 b⋅min^–1^, 70% HR_max_ = 130 ± 5 b⋅min^–1^, 90% HR_max_ = 166 ± 7 b⋅min^–1^.

Women with a regular menstrual cycle lasting 21–35 days were included (Elliott-Sale et al., 2021). They were asked to self-report the first and last day of previous menses and contraceptive use for data analysis and scheduling. All experimental trials were scheduled during the same phase of their menstrual cycle (luteal phase or follicular phase), although the specific phase was not expected to affect study outcomes (Stone et al., 2021). Two of the 4 women completed the experimental trials in the luteal phase of their menstrual cycle. Although phase of menstrual cycle was not confirmed via hormonal assay, based on cycle reporting and rectal temperature (T_re_) it is likely 1 woman completed the 43-min trials in the follicular phase and the remaining trials in the luteal phase. One woman who was using an oral contraceptive [norgestimate (0.25 mg) and ethinyl estradiol (0.035 mg)] was tested in her follicular phase.

For each of the 5 trials, participants were instructed to abstain from consuming alcohol or participating in strenuous exercise during the 24 h before testing. Additionally, participants were asked to report to the laboratory well rested, euhydrated, and having refrained from ingesting non-prescription drugs and caffeine on the day of testing. Pre-testing instructions have been successfully used by our lab previously and adherence was confirmed using a 24-h history questionnaire (Yoder et al., 2023; Mulholland et al., 2023).

Upon arrival, participants provided a urine sample that was analyzed for urine specific gravity (U_SG_) using a digital refractometer (ATAGO PAL-10S digital refractometer, Tokyo, Japan). U_SG_ had to be ≤1.020 for a participant to be considered adequately hydrated (Sawka et al., 2007). Participants whose U_SG_ values were >1.020 were given water to ingest for 20–30 min and then reevaluated. Then participants dressed in padded cycling shorts and a mesh tank top and were equipped with chest strap HR monitor (H10, Polar Electro, Kempele, Finland) that paired with a smartphone application (Polar Beat, version 3.5.0, Polar Electro, Kempele, Finland). Prior to beginning exercise, the Borg 6–20 RPE scale was explained using standardized instructions (Borg and Noble, 1974).

A minimum of 24 h separated control trials from subsequent experimental trials and at least 48 h separated experimental trials from one another. All trials for a given participant took place over ≤8 weeks and each was completed at a similar time of day to control for fluctuations in core body temperature associated with circadian rhythm (Moore-Ede et al., 1983).

### 2.2 Control $\dot{V}$ O_2max_ trial

At the first visit, participants completed a questionnaire about their readiness to participate in exercise and a general health history form. Next, height was measured using a stadiometer (SECA 213, Seca Ltd., Hamburg, Germany) and body mass was measured with a digital scale (Tanita WB-800S, Tanita Corp., Tokyo Japan). Body fat percentage was calculated from the sum of 3 skinfolds (Jackson and Pollock, 1985).

Participants then began a self-selected warm-up for 5–10 min on the cycle ergometer. Next, the GXT started and every 2 min the power output on the cycle ergometer was increased by 25 W until volitional exhaustion was reached or pedal cadence fell below 30 rev⋅min^–1^. $\dot{V}$ O_2_ was measured continuously using open circuit spirometry (Parvo Medics Metabolic Measurement System, model TrueOne 2400, Salt Lake City, UT, United States). $\dot{V}$ O_2max_ was considered as the average of the highest 2 consecutive 30-s values. During the GXT, HR was measured continuously using a smartphone application (Polar Beat, version 3.5.0, Polar Electro, Kempele, Finland) that was integrated with the chest strap and HR_max_ was the highest 1-s value achieved during the test. This value was then used to calculate the THR for the experimental trials. Immediately after completion of the GXT, RPE was obtained from participants (Borg and Noble, 1974). Then, approximately 3–5 min later, a 2-mL blood sample was drawn from a superficial forearm vein into a Vacutainer tube containing EDTA (BD Vacutainer, Becton, Dickinson and Co., Franklin Lakes, NJ, United States) for the measurement of blood lactate in duplicate (YSI 2300 STAT Plus, Yellow Spring Instruments, OH, United States). Researchers provided verbal encouragement to participants during all GXTs.

Twenty min following the GXT, participants completed a $\dot{V}$ O_2max_ plateau verification protocol in which they cycled to volitional exhaustion. Those who completed <1 min of the final stage of the initial GXT performed the verification protocol at the final power output achieved during the initial GXT; those who completed ≥1 min of the final stage of the initial GXT performed the verification protocol at a power output 25 W higher than that achieved during the final stage of the initial GXT (Wingo et al., 2005). To be eligible to continue participation in the study all participants had to exhibit a $\dot{V}$ O_2max_ ≥ 20th percentile for cycle ergometer-based testing for their sex and age (American College of Sports Medicine, 2022).

### 2.3 Experimental trials

At least 24 h following the control trial, participants returned to the laboratory for the first experimental trial. In addition to the procedures outlined under “all trials,” for the experimental trials, participants measured nude body mass and inserted a flexible rectal thermistor 10 cm beyond the anal sphincter for measurement of T_re_. The thermistor was integrated with wireless amplifiers (BioNomadix Wireless SKT Transmitter, Biopac Systems, Inc., Goleta, CA, United States) set to a sampling frequency of 1,000 Hz. T_re_ and ambient temperature were recorded continuously using a data acquisition system (MP150, Biopac Systems, Inc., Goleta, CA, United States) powered by data analysis software (AcqKnowledge 4.2, Biopac Systems, Inc., Goleta, CA, United States). During the trials based on HR, a member of the research team monitored HR and adjusted the workload to maintain HR within 5 b⋅min^–1^ of THR during the entire workout or at 30 W during the recovery intervals if THR was not achievable. During trials based on RPE, the participant adjusted the power output (with the power output concealed) to match the target RPE. The RPE scale was continuously visible to participants throughout the exercise sessions and participants were frequently reminded to adjust resistance to remain at the prescribed intensity. During the HR-based trials, participants were instructed to point to a value on the chart that matched their RPE at the end of the first and fifth work and recovery intervals; the value was verbally confirmed by a member of the research team.

All blood samples taken before, during, and after the experimental trials were drawn from a superficial forearm vein into a Vacutainer containing EDTA for measurement of either lactate concentration, hematocrit (HCT) and hemoglobin (Hb) concentration, or both, as specified in Figure 1. HCT was assessed in triplicate using a microcapillary reader (Model 3201, International Equipment Co., Boston, MA, United States); Hb concentration was assessed in duplicate using a Hb analyzer (HemoPoint H2, EKF Diagnostics, Inc., Boerne, TX, United States). HCT and Hb were then used to calculate plasma volume change (Dill and Costill, 1974). After the last round of HIIT recovery, participants immediately began a GXT in the same manner as during the control trial with no cessation of exercise to determine $\dot{V}$ O_2max_.

Even though the plateau verification procedure for $\dot{V}$ O_2max_ that was completed during the control trials was not completed in the experimental trials, the $\dot{V}$ O_2_ values measured after the 4 experimental trials were still referred to as $\dot{V}$ O_2max_ (instead of $\dot{V}$ O_2peak_). Expressing the values as $\dot{V}$ O_2max_ signified the observed changes were temporary changes in $\dot{V}$ O_2max_, which is consistent with the nomenclature used in other studies involving cardiovascular drift and $\dot{V}$ O_2max_ (Stone et al., 2021; Lafrenz et al., 2008).

#### 2.3.1 15-min trials

Participants entered the environmental chamber and mounted the cycle ergometer. Next, instrumentation was connected and baseline measurements were taken (∼15 min). After baseline measurements, participants completed 1 of the 15-min trials, which included a warm-up and 1 round of HIIT followed by a GXT to determine $\dot{V}$ O_2max_.

#### 2.3.2 43-min trials

For the 43_HR_ and 43_RPE_ trials, skin temperature was measured using 4 iButtons (model no. DS1921H, Embedded Data Systems, KY, United States) taped to each participant’s right upper chest, lateral deltoid, anterior thigh, and lateral calf with elastic therapeutic tape. Skin temperatures from these sites were then used to calculate mean skin temperature ( ${\bar{T}}_{\text{sk}}$ ) using the following equation (Ramanathan, 1964):

$${\bar{T}}_{\text{sk}}=0.3\left(T_{\text{chest}}+T_{\text{delt}}\right)+0.2\left(T_{\text{thigh}}+T_{\text{calf}}\right)$$

where T_chest_, T_delt_, T_thigh_, and T_calf_ are the skin temperatures at the chest, deltoid, thigh, and calf, respectively. Mean body temperature ( ${\bar{T}}_{\text{b}}$ ) was calculated using a weighted average of T_re_ and ${\bar{T}}_{\text{sk}}$ using the following equation (Stolwijk and Hardy, 1966):

$${\bar{T}}_{b}=0.8\left(T_{\text{re}}\right)+0.2\left({\bar{T}}_{\text{sk}}\right)$$

The core-to-skin thermal gradient was calculated as the difference between T_re_ and ${\bar{T}}_{\text{sk}}$ (T_re_– ${\bar{T}}_{\text{sk}}$ ). $\dot{V}$ O_2_ was measured during the first and fifth work interval and the GXT. Metabolic rate was estimated for the first and fifth work intervals using the following equation (Kenny and Jay, 2013):

$$M=(\dot{V}O_{2}\left[\left(\left(\left(\text{RER }‒\;0.7\right)0.3^{‒1}\right)e_{c}\right)+\left(\left(\left(1\;‒\text{ RER}\right)0.3^{‒1}\right)e_{f}\right)\right]60^{‒1}$$

where $\dot{V}$ O_2_ is the rate of oxygen uptake in L⋅min^–1^, *e*_*c*_ = 21,130 J (caloric equivalent per liter of oxygen for carbohydrate oxidation), *e*_*f*_ = 19,630 J (caloric equivalent per liter of oxygen for fat oxidation), and RER is respiratory exchange ratio. The difference between *M* and the external work rate on the cycle ergometer was calculated as the rate of metabolic heat production (*M*–*W)* and expressed in W (Kenny and Jay, 2013).

Next, a flexible catheter was placed into a forearm vein for 2-mL blood sample collection before, during the trial at time points corresponding to the end of the high-intensity bouts (min 12 and 40), and after exercise. Blood lactate concentration was measured at the end of the first and fifth work intervals and upon completion of the GXT. HCT and Hb were measured at baseline and the end of the fifth work interval.

Following the placement of the catheter, participants entered the environmental chamber and mounted the cycle ergometer. The remaining instrumentation was then connected, a 2-mL blood sample was drawn, and other baseline measurements were taken (∼15 min). Participants then began one of the 43-min trials (warm-up, 5 rounds of HIIT, and GXT). At min 12 and 40 participants were asked to report their thermal sensation on a numerical scale (Young et al., 1987). Approximately 20 min after the exercise session, participants were asked to rate the session RPE (Foster et al., 2001).

### 2.4 Data analysis

Mean data were generated on the indicated outcome measures. To test the significance of mean differences in power output, a 2-way [condition × time (work intervals 1 and 5)] repeated measures analysis of variance (ANOVA) was used. Power output was also assessed by comparing the change in power output from the first work interval (min 9–11) to the fifth work interval (min 37–40) between the 43_HR_ and 43_RPE_ trials using a paired samples *t*-test. Paired samples t-tests were also used to evaluate the difference in T_re_, ${\bar{T}}_{\text{sk}}$ , ${\bar{T}}_{\text{b}}$ , T_re_– ${\bar{T}}_{\text{sk}}$ (core-to-skin thermal gradient), and session RPE at the end of the GXT of the 43-min trials.

Baseline data for control and experimental trials were analyzed using a 1-way repeated measures ANOVA. Planned contrasts were performed to compare $\dot{V}$ O_2max_ from each experimental trial to the control trial, using the Bonferroni correction to control for family-wise error rate (α′ = 0.05/number of contrasts). Two-way repeated measures ANOVAs [condition × time (after 15 and after 43 min)] were used to compare $\dot{V}$ O_2max_ and other variables after the GXT after 15 min (1 round of HIIT) to after 43 min (5 rounds of HIIT). To evaluate if $\dot{V}$ O_2max_ decreased by a larger amount depending on the method of exercise prescription (THR or RPE), a paired samples *t*-test compared changes in $\dot{V}$ O_2max_ (from after 15 min to after 43 min).

For hematological variables, 2-way repeated measures ANOVAs [condition × time (work intervals 1 and 5)] were conducted. For other variables, such as HR, T_re_, ${\bar{T}}_{\text{sk}}$ , power output, and $\dot{V}$ O_2_, 2-way repeated measures ANOVAs [condition × time (work intervals 1 and 5) and/or condition × time (recovery intervals 1 and 5)] were conducted. In the event of a significant omnibus test, paired samples t-tests with a Bonferroni-adjusted α level (α′) were used for *post hoc* comparisons as appropriate. Effect sizes (ES) for paired samples t-tests were calculated using the following formula (Lakens, 2013) for Cohen’s *d*_*av*_ (Cohen, 1988), adjusted for positive bias using Hedges’s correction (*g*_*av*_):

$$\text{ES}=\frac{Mean\;difference}{\frac{{SD}_{1}+{SD}_{2}}{2}}\times \left(1-\frac{3}{4\left(n\times 2\right)-9}\right)$$

where, SD_1_ and SD_2_ are the standard deviations of the respective time points or conditions and n is the number of pairs. ES were interpreted as: 0.20 = small, 0.50 = medium, and 0.80 = large, respectively (Caldwell and Cheuvront, 2019; Fritz et al., 2012).

For select variables, the 95% confidence interval (CI) was calculated for the mean difference between conditions (for pairwise comparisons of interest) using a critical t (adjusted, if applicable, to keep the family-wise error rate α at 0.05) in the following formula (Weir and Vincent, 2020):

$$\text{CI}=\text{Mean difference }\pm \;t_{\text{cv}}\left({\text{SE}}_{d}\right),$$

where t_cv_ is the critical t value (adjusted for multiple comparisons, if applicable) and SE_d_ is the standard error of the differences.

For power output, $\dot{V}$ O_2_, and *M*–*W*, the average over the entire interval was used for data analysis; for T_re_ and ${\bar{T}}_{\text{sk}}$ , the average of the final min of the interval was used for data analysis; for HR, both the average over the entire interval and the average of the final min were analyzed. All statistical tests used an α level of 0.05 and analyses were performed using SPSS for Mac v.28.0.0.0 (IBM Corporation, Somers, NY).

## 3 Results

### 3.1 Hydration

Participants were adequately hydrated prior to all trials (mean ± SD, U_SG_ control = 1.005 ± 0.002, 15_HR_ = 1.007 ± 0.005, 15_RPE_ = 1.005 ± 0.004, 43_HR_ = 1.006 ± 0.006, 43_RPE_ = 1.006 ± 0.003; *p* = 0.82). Additionally, pre-exercise body mass was comparable among trials (control = 73.9 ± 7.9 kg, 15_HR_ = 73.9 ± 7.6 kg, 15_RPE_ = 74.4 ± 8.8 kg, 43_HR_ = 74.3 ± 8.9 kg, 43_RPE_ = 74.0 ± 8.0 kg; *p* = 0.68). Percent change in body mass from before to after exercise for each experimental trial was greater in the 45-min vs. 15-min trials (15_HR_ = −0.7% ± 0.4%, 15_RPE_ = −0.6% ± 0.4%, 43_HR_ = −1.3% ± 0.8%, and 43_RPE_ = −1.3% ± 0.8%; *p* = 0.004 for main effect of time). Additionally, percent change in plasma volume pre-to post-HIIT exercise was not different between 43-min trials (43_HR_ = −9.0% ± 3.3%, 43_RPE_ = −9.5% ± 3.9%, *p* = 0.67).

### 3.2 Cardiovascular, work rate, metabolic, and perceptual responses during HIIT exercise

#### 3.2.1 Cardiovascular

**FIGURE 2:**
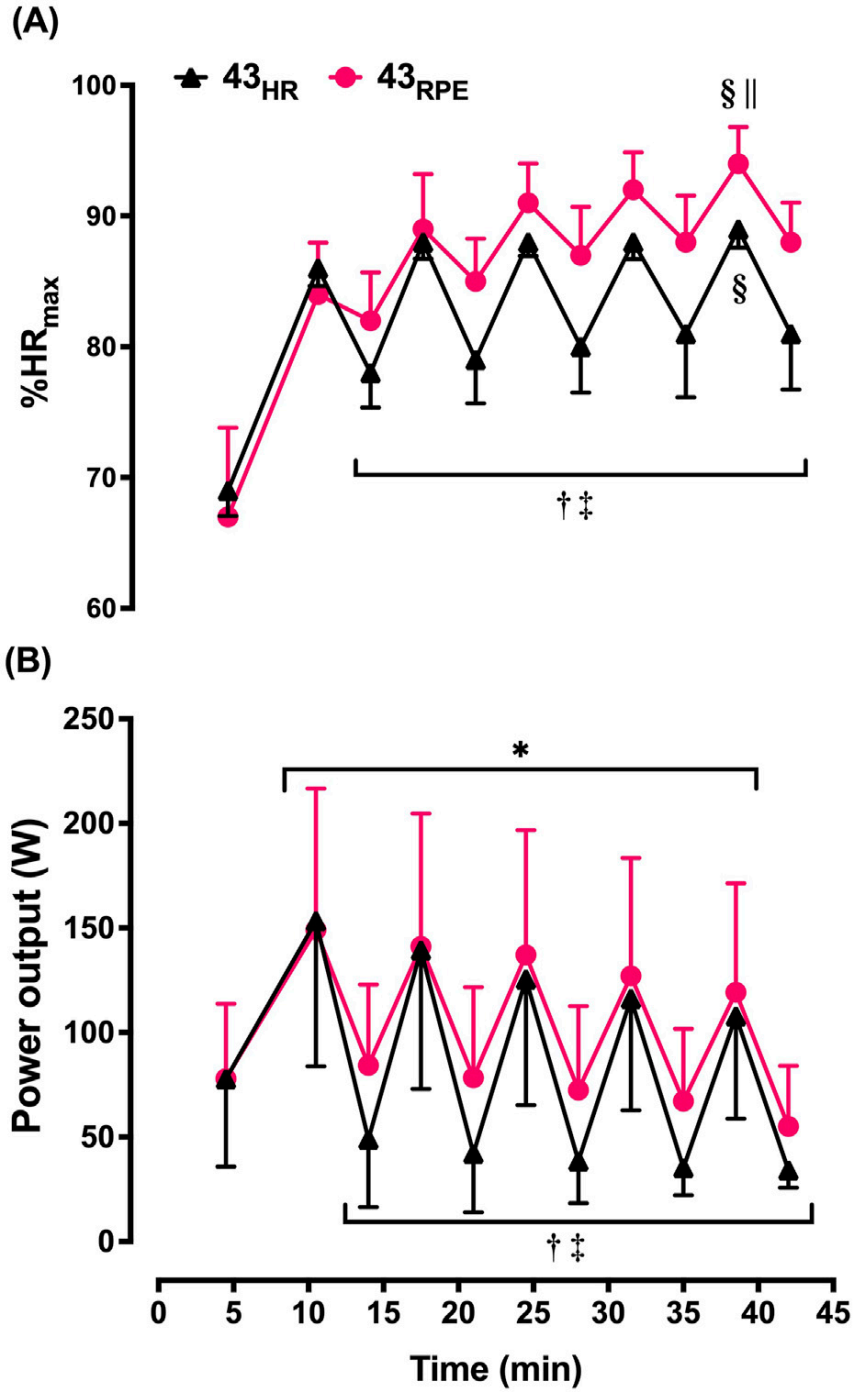
Mean ± SD heart rate (expressed as percentage of maximum [%HR_max_]; **(A)** and power output **(B)** averaged over each interval during the 43-min trials. 43_HR_ = 43-min trial based on target heart rate; 43_RPE_ = 43-min trial based on target rating of perceived exertion. *p < 0.05 main effect of time during work intervals; ^†^ *p* < 0.05 main effect of time during recovery intervals; ^‡^ *p* < 0.05 main effect of condition during recovery intervals; ^§^ *p* < 0.05 compared with work interval 1 of the given condition; ^||^ *p* < 0.05 compared with heart rate-based trial during the same work interval.

As designed, during the work intervals of the HR-based trial, HR during the final min did not increase from the first to fifth interval and THR was achieved (Table 1; Figure 2). In contrast, during 43_RPE_, HR increased by 12 b⋅min^–1^ from the first to fifth work interval. During the final min of recovery intervals across both 43-min trials, HR increased by 11 b⋅min^–1^ from the first to fifth recovery interval and HR was 13 b⋅min^–1^ higher during the RPE-based trial. HR during the final min of the first and fifth recovery intervals increased from 72% to 78% HR_max_ in the HR trial and from 79% to 85% HR_max_ in the RPE trial (*p* < 0.001 for main effect of time; *p* = 0.006 for main effect of condition). Similar patterns were observed for %HR_max_ averaged over the entire work and recovery intervals and are shown in Figure 2A.

**TABLE 1:** Responses during the first (1) and fifth (5) work and recovery intervals.

Work	HR		RPE		Interaction
	Interval 1	Interval 5	Interval 1	Interval 5	*P*
∆Power output (%)	—	−30 ± 10	—	−18 ± 18	—
∆Power output (W)	—	−46 ± 29	—	−30 ± 10 ^||^	—
Average HR (b⋅min^–1^)	159 ± 6	165 ± 8^‡^	156 ± 8	173 ± 4^‡§^	0.01
Final min HR (b⋅min^–1^)	168 ± 6	167 ± 7	164 ± 6	176 ± 5^‡§^	<0.001
$\dot{V}$ O_2_ (L·min^–1^)	2.3 ± 1.1	2.0 ± 0.8	2.2 ± 1.0	2.2 ± 0.9	0.16
%Control $\dot{V}$ O_2max_	72 ± 5	64 ± 8	71 ± 7	71 ± 11	0.07
Blood lactate (mmol·L^–1^)^*^	3.2 ± 1.0	2.5 ± 1.5	2.8 ± 1.2	3.2 ± 2.0	0.31
RPE	16 ± 2	16 ± 1	—	—	—
*M* – *W* (W)	622 ± 290	603 ± 287	620 ± 300	673 ± 312	0.04
Recovery					
∆Power output (%)	—	−18 ± 24	—	−33 ± 14	—
Average HR (b⋅min^–1^)^*‡^	144 ± 10	150 ± 12	152 ± 8	163 ± 5	0.08
Final min HR (b⋅min^–1^)^*‡^	133 ± 11	144 ± 15	147 ± 9	157 ± 6	0.91
$\dot{V}$ O_2_ (L·min^–1^)^*†a^	1.6 ± 0.7	1.3 ± 0.4	1.9 ± 0.8	1.6 ± 0.6	0.83
%Control $\dot{V}$ O_2max_^*†a^	51 ± 6	43 ± 6	61 ± 10	53 ± 8	0.90
RPE	11 ± 2	12 ± 2	—	—	—
Thermal sensation^*^	6.0 ± 1.0	7.0 ± 0.5	6.0 ± 0.5	7.0 ± 0.5	0.44
${\bar{T}}_{\text{sk}}$ (°C)^*^	36.3 ± 0.4	36.7 ± 0.6	36.4 ± 0.3	36.9 ± 0.5	0.40
T_re_ – ${\bar{T}}_{\text{sk}}$ (°C)^*^	1.0 ± 0.4	1.3 ± 0.4	0.9 ± 0.3	1.3 ± 0.3	0.78
∆T_re_ (°C)	—	0.7	—	0.8	0.04
T̅_b_ (°C)	37.1 ± 0.3	37.8 ± 0.4^‡^	37.1 ± 0.3	37.9 ± 0.4^‡^	0.03

HR, heart rate; RPE, rating of perceived exertion; ∆Power output = change in power output from the first to fifth interval; %HR_max_, percent of maximal HR, averaged over the interval; $\dot{V}$ O_2_ = oxygen uptake; RPE, rating of perceived exertion; *M*–*W* = rate of metabolic heat production; T_re_ = rectal temperature during the final min of intervals 1 and 5; ∆T_re_ = change in rectal temperature from the final min of interval 1 to the final min of interval 5; ${\bar{T}}_{\text{sk}}$ = mean skin temperature during the final min of intervals 1 and 5; T_re_– ${\bar{T}}_{\text{sk}}$ = core-to-skin thermal gradient during the final min of intervals 1 and 5,^a^ Recovery $\dot{V}$ O_2_ data from interval 4 were used in both conditions for 1 participant.

^*^ *p* < 0.05 for main effect of time; ^†^ *p* < 0.05 for main effect of condition; ^‡^ *p* < 0.05 compared with interval 1 within the same condition; ^§^ *p* < 0.05 compared with HR-based trial during the same interval; ^||^ *p* < 0.05 for paired samples *t*-test between HR-based and RPE-based trials.

During 43_HR_, 4 participants were able to reach THR during the first recovery interval, 3 in the second, and the same 2 participants for the final 3 recovery intervals. During the 43_RPE_ trial, 2 participants cycled at the minimum 30 W for the final 2 intervals. Two participants reached or surpassed the HR_max_ observed in the control trial during the HIIT portion of the 43_RPE_ trial.

#### 3.2.2 Power output

Across both 43-min trials, power output had to be lowered by 38 W (ES = 0.59) between the first and fifth work intervals to maintain the target intensity, but conditions were not statistically different (Table 1; Figure 2B). Likewise, the *t*-test comparing the change score between the first and fifth work interval for 43_HR_ (−46 ± 29 W) and the change score between the first and fifth work interval for 43_RPE_ (−30 ± 28 W) was not statistically significant [mean difference (MD) = 16 ± 36 W; 95% CI for MD = −45, 15; ES = 0.53 ], but the magnitude of difference between these change scores was moderate. During the recovery intervals power output was 22 W lower during the fifth interval across both conditions (ES = 0.79) and 28 W (ES = 1.00) lower in the HR-based trial across both time points.

#### 3.2.3 Metabolic and perceptual responses

In the work intervals, absolute $\dot{V}$ O_2_ was not different over time even though the experimental conditions were based on different methods of gauging exercise intensity. In contrast, in the recovery intervals, absolute $\dot{V}$ O_2_ was lower during the HR-based trials (*p* = 0.004) and decreased over time in both conditions (*p* = 0.02). Thermal sensation increased from 6.0 to 7.0 from the end of the first work interval to the final work interval across both 43-min trials (Table 1). Likewise, session RPE was similar between 43_HR_ (8 ± 1) and 43_RPE_ (9 ± 1) (*p* = 0.44).

### 3.3 Thermoregulatory responses to HIIT exercise

**FIGURE 3:**
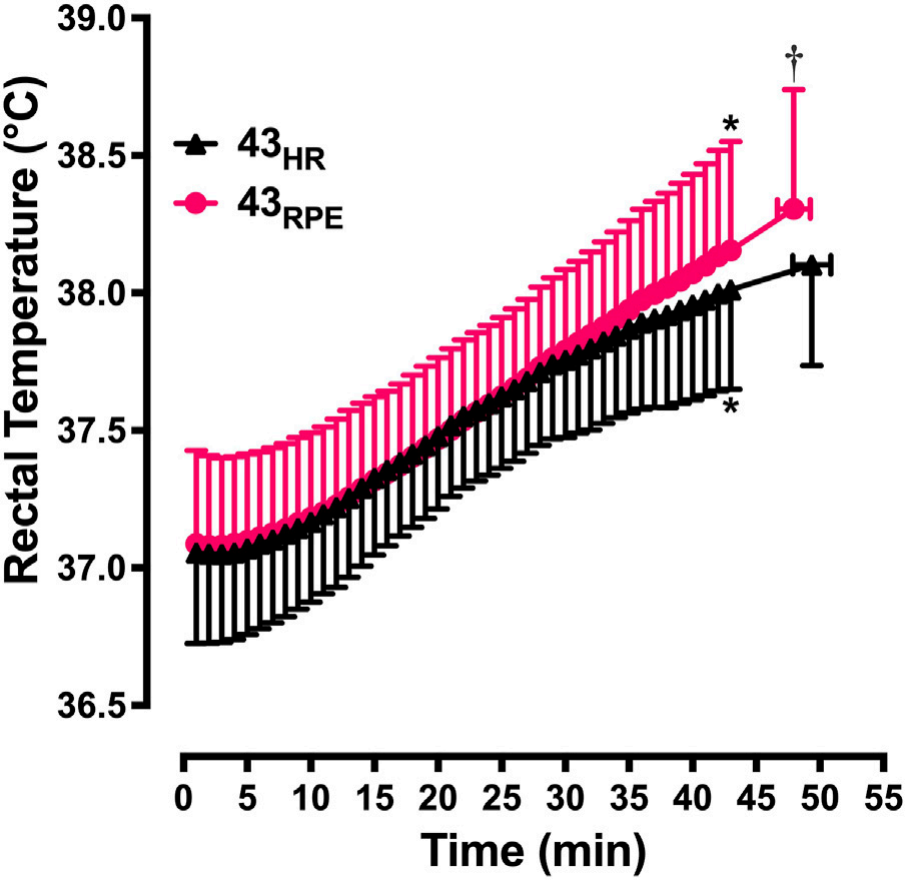
Mean ± SD rectal temperature from the start of exercise to the end of the graded exercise test. 43_HR_ = 43-min trial based on target heart rate; 43_RPE_ = 43-min trial based on target rating of perceived exertion. *p < 0.05 compared to min 15 of the same condition; ^†^ *p* < 0.05 compared to 43_HR_ at maximum effort.

Baseline T_re_ was not different among the 4 experimental trials (15_HR_ = 37.3°C ± 0.3°C, 15_RPE_ = 37.2°C ± 0.3°C, 43_HR_ = 37.1°C ± 0.3°C, 43_RPE_ = 37.1°C ± 0.3°C, *p* = 0.19). *M–W* increased over time in 43_RPE_ and decreased over time in 43_HR_. Nonetheless, T_re_ increased by a comparable amount between experimental conditions so that T_re_ at the end of the final recovery interval was not different between conditions (MD = 0.2 ± 0.2; 95% CI for MD = −0.05, 0.46; ES = 0.36; Figure 3). T_re_ was also similar between conditions at the end of the first recovery interval. By the end of the GXT in the 43-min trials, T_re_ was higher following the RPE trial compared to the HR trial (*p* = 0.03). Across both experimental conditions, ${\bar{T}}_{\text{sk}}$ increased from the end of the first to final recovery interval by ≈ 0.4°C. The core-to-skin thermal gradient (T_re_– ${\bar{T}}_{\text{sk}}$ ) followed a similar pattern and increased by 0.3°C in both trials. ${\bar{T}}_{\text{b}}$ increased by 0.6°C in 43_HR_ and by 0.8°C in 43_RPE_ from the end of the first to final recovery interval.

### 3.4 Maximal responses

**FIGURE 4:**
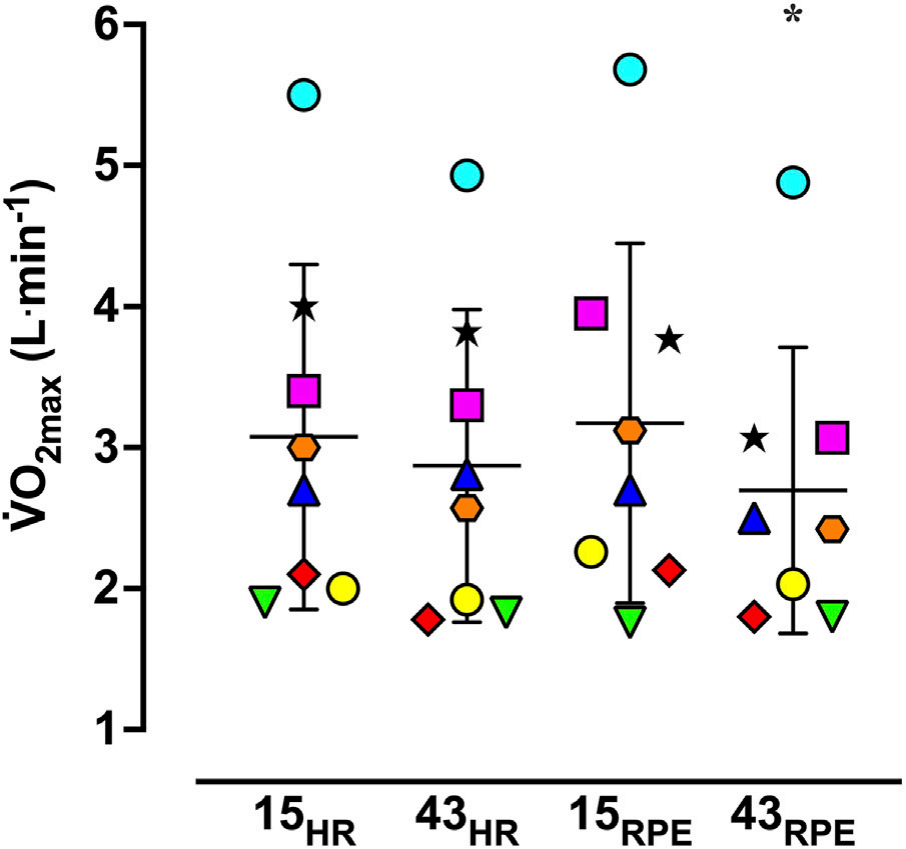
Vertical scattergram of maximal oxygen uptake ( $\dot{V}$ O_2max_) during the experimental trials. 15_HR_ = 15-min trial based on target heart rate; 15_RPE_ = 15-min trial based on target rating of perceived exertion; 43_HR_ = 43-min trial based on target heart rate; 43_RPE_ = 43-min trial based on target rating of perceived exertion. Symbols represent data from individual participants and horizontal bars and accompanying error bars represent mean ± SD. *p < 0.05 compared to 15_RPE_.

Maximal responses are shown in Table 2 and Figure 4. Planned comparisons between control $\dot{V}$ O_2max_ (3.2 ± 1.2 L⋅min^–1^) and $\dot{V}$ O_2max_ after each experimental trial did not reveal any differences [(α′ = 0.0125) *p* = 0.58 for 15_HR_; *p* = 0.52 for 15_RPE_; *p* = 0.014 for 43_HR_; *p* = 0.014 for 43_RPE_]. $\dot{V}$ O_2max_ decreased 15.6% between 15_RPE_ and 43_RPE_ (MD = 0.5 ± 0.3 L⋅min^–1^; 95% CI for MD = 0.08, 0.87; ES = 0.41; Figure 4), but it was not different over time during the HR-based trials [MD = −0.2 ± 0.2 L⋅min^–1^, 95% CI for MD = −0.05, 0.47; ES = 0.16). Furthermore, the change score (15-min value minus 43-min value) in $\dot{V}$ O_2max_ for RPE-based trials was greater than HR-based trials (MD = 0.3 ± 0.3 L⋅min^–1^, 95% CI for MD = 0.01, 0.52; ES = 1.13). However, $\dot{V}$ O_2_ was not different between overtime or between conditions (*p* = 0.16 for interaction effect). During 43_HR_, because $\dot{V}$ O_2max_ and absolute $\dot{V}$ O_2_ during HIIT exercise did not change over time, relative intensity ( $\dot{V}$ O_2_ expressed as a percentage of $\dot{V}$ O_2max_ at the specified time point) was maintained from work interval 1 (72% ± 5% $\dot{V}$ O_2max_) to work interval 5 (69% ± 8% $\dot{V}$ O_2max_, *p* = 0.17). Because $\dot{V}$ O_2max_ decreased during 43_RPE_ and absolute $\dot{V}$ O_2_ did not change over time, relative intensity increased by 11 percentage units from work interval 1 (70% ± 10% $\dot{V}$ O_2max_) to work interval 5 (81% ± 11% $\dot{V}$ O_2max_, *p* = 0.006; *p* = 0.002 for interaction effect), and it was 11.5 percentage units higher on average during work interval 5 in 43_RPE_ *versus* 43_HR_ (*p* = 0.01).

**TABLE 2:** Maximal responses during a graded exercise test following 15 min (after 1 work and recovery interval) and 43 min (after 5 work and recovery intervals) of high-intensity interval training exercise in a hot environment using heart rate or rating of perceived exertion to prescribe exercise intensity.

Variable	Trial				Interaction
	15_HR_	43_HR_	15_RPE_	43_RPE_	*P*
$\dot{V}$ _E_ (STPD, L·min^-1^)^*^	84.9 ± 24.4	80.0 ± 21.6	88.6 ± 24.9	74.1 ± 20.1	0.08
$\dot{V}$ O_2_ (mL·kg^-1^·min^-1^)	41.0 ± 13.7	38.0 ± 12.2	41.9 ± 13.7	35.9 ± 11.2^‡^	0.020
$\dot{V}$ O_2_ (L·min^-1^)	3.1 ± 1.2	2.8 ± 1.3	3.2 ± 1.3	2.7 ± 1.0^‡^	0.041
Power output (W)	194 ± 73	172 ± 69^‡^	197 ± 78	141 ± 72^‡§^	0.028
RER	1.03 ± 0.04	1.02 ± 0.05	1.06 ± 0.06	0.95 ± 0.07^§^	0.029
RPE	20 ± 1	20 ± 1	20 ± 1	20 ± 0	0.35
HR (b·min^-1^)	187 ± 7	185 ± 5	187 ± 8	187 ± 6	0.21
Blood lactate (mmol·L^-1^)^*^	4.9 ± 1.4	3.7 ± 1.1	5.4 ± 1.4	3.5 ± 1.5	0.31
Test duration (min)^*^	7.8 ± 1.4	6.4 ± 1.5	8.8 ± 1.3	5.0 ± 1.3	0.06
${\bar{T}}_{\text{sk}}$ (°C)	—	36.6 ± 0.6	—	36.9 ± 0.6	—
T_re_ (°C)^*†^	37.8 ± 0.3	38.1 ± 0.4	37.8 ± 0.3	38.3 ± 0.4	0.10
T_re_ – ${\bar{T}}_{\text{sk}}$ (°C)	—	1.5 ± 0.5	—	1.4 ± 0.5	—
${\bar{T}}_{\text{b}}$ (°C)	—	37.8 ± 0.4	—	38.0 ± 0.4 ^||^	—

15_HR_, 15-min trial based on target heart rate; 15_RPE_, 15-min trial based on target rating of perceived exertion; 43_HR_, 43-min trial based on target heart rate; 43_RPE_, 43-min trial based on target rating of perceived exertion; $\dot{V}$ _E_ = minute ventilation; $\dot{V}$ O_2_ = oxygen uptake; RER, respiratory exchange ratio; RPE, rating of perceived exertion; HR, heart rate; ${\bar{T}}_{\text{sk}}$ = mean skin temperature; T_re_ = rectal temperature; ${\bar{T}}_{\text{b}}$ = mean body temperature.

^*^ *p* < 0.05 for main effect of time; †*p* < 0.05 main effect of condition; ^‡^p < 0.05 compared with interval 1 within the same condition; §p < 0.05 compared with HR-based trial during the same interval; ||p < 0.05 for paired samples *t*-test between HR, and RPE.

For the 43-min trials, the maximal power output achieved during the GXT was lower compared to the respective 15-min trial. Additionally, the maximal power output during the GXT was 31 W lower in the 43_RPE_ trial *versus* the 43_HR_ trial. T_re_ and ${\bar{T}}_{\text{b}}$ were both 0.2°C higher on average (ES = 0.47 for both) upon completion of the GXT following 43_RPE_ compared to 43_HR_; however, ${\bar{T}}_{\text{sk}}$ and the core-to-skin thermal gradient (T_re_– ${\bar{T}}_{\text{sk}}$ ) were not different between conditions at maximal effort in the 43-min trials.

## 4 Discussion

The purpose of this study was to evaluate work rate adjustments and thermal and cardiovascular strain using two simple methods of exercise prescription, THR and target RPE, to prescribe HIIT in the heat. A secondary purpose was to evaluate changes in aerobic capacity ( $\dot{V}$ O_2max_) before (1 round of HIIT equal to 15 min of exercise) and after (following 5 rounds of HIIT equal to 43 min of exercise) cardiovascular drift is known to occur. The primary outcome was that work rate decreased from the first to the fifth work interval in both conditions, but by a non-significant, yet 53% larger amount during 43_HR_ (46 W) compared to 43_RPE_ (30 W). The moderately smaller reduction in work rate during 43_RPE_ did not result in differences in T_re_ over time between the two 43-min trials, except upon completion of the GXT. However, as hypothesized, participants experienced increased cardiovascular strain during the 43_RPE_ trial; HR was 9 b⋅min^–1^ higher during the final work interval and 13 b⋅min^–1^ higher during the first and final recovery intervals. Furthermore, a greater reduction in maximal aerobic capacity was observed following 43_RPE_ compared to 43_HR_.

The range of decreases in work rate from the first to final work interval (43_RPE_ = 18% and 43_HR_ = 30%) were comparable to what others have observed during HIIT in a temperate environment using THR (21%) (Morales-Palomo et al., 2017), during 45 min of continuous exercise in the heat using THR (37%) (Wingo and Cureton, 2006b), and during 30 min of continuous exercise in the heat using target RPE (≈27%) (Tucker et al., 2006). While work rate was not statistically different during the work intervals, the 16 W (ES = 0.53) greater reduction in work rate during 43_HR_ may be practically meaningful. However, unlike our results where work rate was lower during recovery intervals of 43_HR_, no differences in running speed were observed during work or recovery intervals when using RPE compared to THR to prescribe exercise intensity during a 20-min treadmill walking/running HIIT session in a temperate environment (Ciolac et al., 2015). Still others have found lower intensities using RPE compared to THR during interval training (Aamot et al., 2014) and during continuous exercise (Shea et al., 2022) in cardiac rehabilitation patients in temperate environments. Taken together, it appears that findings related to work rate adjustments during HIIT exercise based on THR and target RPE are equivocal. It is likely the heat stress in the current study contributed to the variability of findings in the literature. Differences could also be attributed to variations in exercise protocol, mode and duration, or participant characteristics.

We predicted work rate and thermal strain would be greater during 43_RPE_ *versus* 43_HR_, but statistically higher work rates were only observed during the recovery intervals of 43_RPE_, and did not result in increased thermal strain during the HIIT protocol. During the HIIT sessions, T_re_, ${\bar{T}}_{\text{sk}}$ , and the core-to-skin thermal gradient (T_re_– ${\bar{T}}_{\text{sk}}$ ) were similar between conditions. The increase in *M–W* in 43_RPE_ was apparently not large enough to result in differences in T_re_ between conditions. Maxwell et al. (2008) observed a higher T_re_ during a sprint interval exercise session (20 × 5-s sprints interspersed with 110-s recovery) in the heat with higher *versus* lower recovery intensities. Differences in the ratio of work to recovery intervals, as well as the intensities used, may explain the differences between the results of the present study and those of Maxwell et al. (2008).

Although the elevated work rate during the recovery intervals of the RPE-based trial did not result in increased thermal strain, cardiovascular strain was greater during 43_RPE_ as indicated by an ≈ 5% higher HR averaged over the fifth work interval and in the final min of the fifth work interval. An increase in HR over time during interval training was observed in temperate environments when work rate was held constant (Thomas et al., 2020), when work rate was self-selected during intervals of 4 or 8 min (Fennell and Hopker, 2021), and in temperate and hot environments when maximal sprint intervals were performed (Maxwell et al., 2008). Fennell and Hopker (2021) manipulated recovery intensity during a similar HIIT protocol (6 × 4 min with 2 min recovery in a temperate environment) where participants recovered at 80% or 110% of the power output corresponding to their lactate threshold. Unlike our results, the different recovery intensities did not affect HR during the work intervals, but during the recovery intervals HR was 7 b⋅min^–1^ higher during the 110% compared to 80% power output of their lactate threshold, respectively. Similarly, the difference in HR between conditions was unlike the findings of Ciolac et al. (2015) and Johnson et al. (2017) who observed similar HR when using THR and RPE to prescribe running intensity in temperate indoor and outdoor environments at varying exercise intensities. It appears that heat stress may alter the relationship between HR and RPE that is observed in temperate environments.

The progressive increase in HR (and accompanying decrease in stroke volume) during continuous exercise in the heat has been shown to be associated with decreased maximal aerobic capacity (Wingo and Cureton, 2006b; Lafrenz et al., 2008). As such, the ∼2.5 times greater decrease in $\dot{V}$ O_2max_ in 43_RPE_ compared to 43_HR_ is consistent with our hypothesis. The 16% reduction in $\dot{V}$ O_2max_ between 15_RPE_ and 43_RPE_ is similar to reductions observed following 45 min of continuous exercise in the heat during cycling (13%) and running (15%) (Wingo et al., 2020). The magnitude of change following the RPE trial is also comparable to the decline observed during a repeated time trial performance (×4 16.5 min with 5 min active recovery) in the heat; $\dot{V}$ O_2max_ was 97% of the control $\dot{V}$ O_2max_ at the end of the first time trial and decreased to 85% at the end of the final time trial (Périard and Racinais, 2015). However, in this same study, during each time trial, participants maintained the same relative intensity (% $\dot{V}$ O_2max_), based on the $\dot{V}$ O_2max_ at that moment, despite the decreasing maximal aerobic capacity (Périard and Racinais, 2015). In the present study, relative intensity was maintained during 43_HR_ (∼71% $\dot{V}$ O_2max_), but during 43_RPE_ it increased by 11 percentage units to 81% $\dot{V}$ O_2max_ from the first to last work interval. The non-significant and small (6.5%, ES = 0.16) decline between 15_HR_ and 43_HR_ is comparable to the 7.5% reduction Wingo and Cureton (2006b) observed following 45 min of continuous exercise in the heat using THR to prescribe exercise intensity. These results indicate that when HR is allowed to drift upwards during the work intervals of HIIT exercise, cardiovascular drift accumulates and is accompanied by declines in maximal aerobic capacity.

Although work rate and $\dot{V}$ O_2_ were not statistically different during the work intervals in the HR-*versus* RPE-based trials, the method of exercise prescription had a moderate to large effect on work rate during the work and recovery intervals, respectively, which explains why HR was elevated in the final work interval of 43_RPE_. The higher work rates sustained in 43_RPE_ drove *M–W* upward. Since heat strain results in tachycardia from increased sympathetic nervous system activity (Gorman and Proppe, 1984) and catecholamine release (Kim et al., 1979), as well as direct effects of heat increasing sinoatrial node firing (Bolter and Atkinson, 1988), the increase in *M–W* could explain exacerbated cardiovascular strain over the course of the 43_RPE_ HIIT session.

As mentioned, aerobic capacity decreased over twice as much following the RPE trial. We speculate the greater *M–W* in this trial resulted in a larger peripheral displacement of blood volume to the skin for heat dissipation. This peripheral displacement of blood volume, combined with the higher HR at the end of the final round of HIIT, could have corresponded to a lower stroke volume (Coyle and Gonzalez-Alonso, 2001; Turkevich et al., 1988; Rowell et al., 1966; Rowell et al., 1969; Wingo et al., 2012). If this lower stroke volume persisted during maximal exercise, it could explain the decrease in $\dot{V}$ O_2max_.

Although both conditions resulted in large declines in work rate, participants were able to complete the entire HIIT protocol followed by a GXT in the heat. Exploring other methods or strategies for intensity prescription of HIIT in the heat may be beneficial for maintaining work interval intensity. Manipulating recovery intensity (such as using passive recovery instead of active recovery) may be one way to preserve work rate during the work intervals based on RPE.

### 4.1 Limitations

A limitation of using RPE to prescribe exercise intensity is the different interpretations of the scale. The RPE scale was explained to participants using standardized instructions and they were instructed to complete the work intervals at an RPE of 17 which meant adjusting the resistance up or down to elicit the prescribed RPE. Nonetheless, it appears 1 participant paced themselves and increased power output from the first to final work interval in the 43_RPE_ trial. This participant preserved their $\dot{V}$ O_2max_ compared to the 15-min trial while the remaining 7 participants experienced an 18% decline (0.6 L min^–1^) in $\dot{V}$ O_2max_ on average between the 15-min and 43-min trials. The participant who started slower may have employed teleoanticipation whereby exercise intensity is regulated based on the anticipated endpoint of the exercise session (Ulmer, 1996), although it is unclear why only 1 person may have adopted this strategy. The large range in adjustments during the RPE trial could be partially attributed to the difference in fitness levels of participants; however, this should not have affected the results because of the repeated measures study design. RPE is easy to use for exercise prescription and it can be practical for prescribing HIIT (Buchheit and Laursen, 2013), but the range of work rate adjustments during the work intervals of 43_RPE_ (−69 W to +19 W) highlights the challenges in using RPE.

Another challenge with using RPE to prescribe intensity during exercise in the heat is the disassociation between the prescribed intensity and the HR response observed during recovery intervals. For instance, participants were instructed to cycle at an RPE of 12 during recovery, which is considered a moderate intensity corresponding to 64%–74% HR_max_ (Garber et al., 2011). However, based on %HR_max_, participants exercised at a vigorous intensity (85% HR_max_) during the final recovery interval (Garber et al., 2011). Even during the first interval of recovery, based on HR, participants were at a vigorous intensity (79% HR_max_). HR was elevated to such an extent that the %HR_max_ during the last 3 recovery intervals of 43_RPE_ were about the same as the work intervals of the 43_HR_ trial (Figure 2A). Furthermore, 2 participants achieved or surpassed the HR_max_ observed during the control trial during the submaximal HIIT portion of 43_RPE_. Using THR in the heat also proved problematic for prescribing intensity during recovery intervals because most participants were unable to achieve the THR and instead cycled at 30 W.

Despite the limitations of using RPE and THR to prescribe intensity in the present study, both resulted in the exercise intensity being attainable across the varying fitness levels of the participants.

### 4.2 Conclusion

Using target RPE and THR to prescribe HIIT exercise in the heat resulted in considerable declines in work rate during the work intervals, and lower work rates were needed to maintain THR compared to target RPE during the recovery intervals. The higher power output sustained in 43_RPE_ recovery intervals corresponded to elevated cardiovascular strain during both work and recovery intervals, as well as a greater decline in $\dot{V}$ O_2max_ over time. The non-significant, moderately smaller reduction in work rate from the first to fifth work interval of 43_RPE_ may have also contributed to the increased cardiovascular strain observed. Although with both methods of exercise prescription reductions in work rate were necessary to maintain the target intensity, all participants (regardless of varying fitness levels; e.g., $\dot{V}$ O_2max_ range: 1.9–5.4 L ⋅ min^‒1^) were able to complete the HIIT exercise protocol, which as our pilot testing indicated, would not have been possible if work rate adjustments had not been made. If total energy expenditure is the goal of the exercise session and magnitude of cardiovascular strain is not important, using RPE to prescribe intensity during HIIT exercise may be preferable. Using THR may be preferable if cardiovascular strain is a concern; however, this may limit the training stimulus since larger declines in work rate are necessary.

## Data Availability

The raw data supporting the conclusions of this article will be made available by the authors, without undue reservation.
